# Intracranial Venous Sinus Stenting: A Review of Idiopathic Intracranial Hypertension and Expanding Indications

**DOI:** 10.7759/cureus.4008

**Published:** 2019-02-04

**Authors:** Lekhaj C Daggubati, Kenneth C Liu

**Affiliations:** 1 Neurosurgery, Penn State Health Milton S. Hershey Medical Center, Hershey, USA; 2 Neurosurgery, Penn State Milton Health S. Hershey Medical Center, Hershey, USA

**Keywords:** idiopathic intracranial hypertension, venous sinus stenting, pseudotumor cerebri

## Abstract

Idiopathic intracranial hypertension (IIH) is a functionally limiting disorder secondary to increased intracranial pressures (ICPs) with a prevalence of one per 100,000 persons. It is estimated to cost >$400 million per year in productively. Symptoms classically consist of chronic headaches, papilledema, and visual loss. The pathophysiology is unknown but postulated to involve increased resistance to cerebrospinal fluid (CSF) absorption. Traditional treatments involve weight loss, acetazolamide, CSF diversion, or optic nerve fenestration. More recent technology has allowed exploration of venous sinus stenosis. Through venous sinus stenting (VSS), the ICPs and venous sinus pressures decrease. After treatment, >75% exhibit an improvement in headaches, ~50% improvement in tinnitus, and ~50 % improvement in ophthalmologic testing. Complications are rare but involve stent stenosis, femoral pseudoaneurysm, and hemorrhages. Future studies will look into controlled studies for VSS as well as expansion to other venous structures of the intracranial circulation.

## Introduction and background

Introduction

Idiopathic intracranial hypertension (IIH), also known as pseudotumor cerebri and benign intracranial hypertension, is a disorder characterized by persistently increased intracranial pressure (ICP) without an otherwise distinguishable pathology. It was first described by a German physician, Heinrich Quincke as Meningitis Serosain 1893, but not until the studies of a Davidoff and Dyke in 1936 was it characterized with a normal ventriculogram [1-2]. Foley was the first to label it as benign intracranial hypertension in 1955 after decades of various pseudonyms [3]. Since then, this disorder has been increasingly recognized with an evolving criterion. The overall population incidence is approximately 0.9-1.07 per 100,000 persons but significantly increases to 15-19 per 100,000 in overweight women aged 20-44 years (>20% of ideal body weight) in North America. A 2011 United Kingdom study reported an incidence of 1.56 per 100,000 people and 11.9 per 100,000 obese women [4-5]. This is primarily a disorder afflicting women; men only constitute approximately 10% of the population with IIH. As it is a disorder inflicted at the peak of financial productivity, the economic cost of IIH is estimated to exceed $444 million in the USA alone and expected to increase in the coming years [6]. 

The classic triad includes chronic headaches, papilledema, and visual loss in the absence of other neurological signs, ventriculomegaly, and intracranial lesions on neuroimaging. The one exception is the occasional abducens nerve palsy noted in severe cases [3]. In addition to the trial, other symptoms include pulse-synchronous tinnitus, diplopia, and progressive visual loss. Headache is the most widely reported symptom with a prevalence of 93%. It is described routinely as severe, daily pulsatile headaches. Other symptoms, in decreasing order of prevalence, are visual obscurations (68%), pulse synchronous tinnitus (58%), photopsia (54%), retrobulbar pain (44%), diplopia (38%), and visual loss (30%) [7].

The neurologic signs are papilledema, abducens nerve palsy, visual acuity worsening, and visual field loss. Papilledema is often considered a defining sign of IIH with an incidence of 95% [8]. Though labeled as "benign", this disorder can cause significant morbidity if left untreated. Approximately 10% to 25% of patients develop visual loss secondary to chronic papilledema from the damage to the optic disc [7,9]. Visual acuity is typically normal in patients with papilledema unless the papilledema persists for a long time. Though patients primarily focus on their headaches, visual loss remains the most feared complication. Recent studies have demonstrated a direct relationship between severity of papilledema and visual acuity loss and visual field eccentricity. The visual field loss manifests as an enlargement of the physiologic blind spot and loss of an inferonasal portion of the visual field [10]. Abducens nerve palsy is found in 10% to 20% of patients [11]. The pathophysiology of these signs is beyond the scope of this review. 

**Figure 1:**
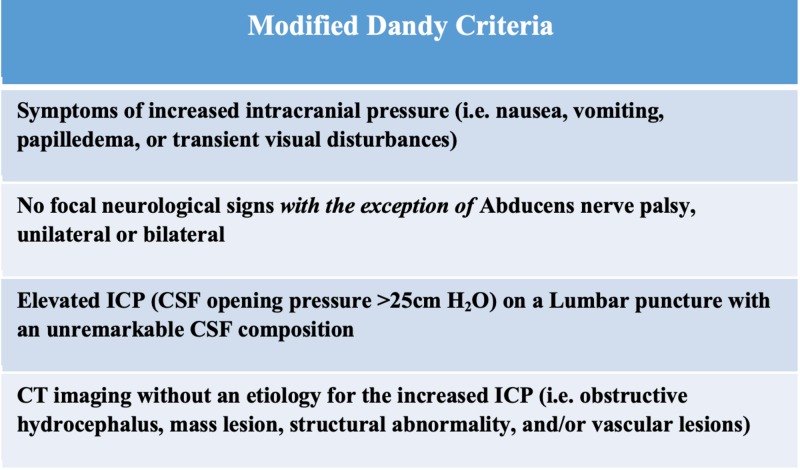
The Modified Dandy Criteria was a clinical criterion develop to distinguish idiopathic intracranial hypertension

The modified Dandy criteria are the most widely accepted criteria (Figure 1). It requires 1) classic symptoms, 2) signs for only elevated ICP, 3) cerebrospinal fluid opening pressure of >25cm H_2_O from a lumbar puncture in a lateral decubitus position, 4) normal CSF composition, and 5) exclusion of a structural abnormality through advanced neuroimaging (magnetic resonance imaging [MRI], contrast-enhanced computed tomography [CT], and/or MR venography) [12]. 

With the rapid advancement in imaging modalities, various signs on MRI have been associated with IIH and these assist in the establishment of IIH. These features include an empty sella turcica filled with CSF, deformed pituitary gland (occupying less than 50% of the fossa with an upper concavity), slit-like ventricles, tight sulcal and cisternal subarachnoid spaces, flattening of the posterior aspect of the optic globe with outward convexity of the globe in severe cases, distension of the optic nerve sheath with a width greater than 2 mm, vertical tortuosity of the optic nerve, and enhancement of the optic nerve. Agid et al. examined the sensitivity and specificity of these signs in 2006. The most sensitive signs were optic nerve sheath distention, pituitary compression, and globe flattening at 66.7%, 53.3%, and 43.3%, respectively, but the most specific were slit-like ventricles, optic nerve enhancement, and empty sella at 100%, 98.2%, and 94.6%, respectively [13]. Though they do not replace the modified Dandy criteria, the radiologic findings are slowly improving the confidence of IIH diagnosis. 

Differential diagnosis

As it is idiopathic, it is vital to exclude any other sources of neurological disease. There are various mimickers of IIH including structural, endocrine, nutritional, medicinal, and genetic disorders. Intracranial hypertension can be caused by any disorder that occludes CSF drainage (meningitis, venous sinus thromboses, and right heart failure), increases blood outflow (arteriovenous malformation or Dural arteriovenous fistulas), scars arachnoid granulations (meningitis, hemorrhage, sarcoidosis), or abnormal structures (mass lesion). Though not fully understood, steroid withdrawal, Addison's disease, and major endocrinological disorders have been linked to elevated ICPs [7].

Pathophysiology

Throughout the centuries, the pathogenesis of IIH has been elusive. Numerous etiologies have been proposed to attempt and describe the phenomenon of idiopathic elevated ICPs. A long list of risk factors has been collected through the decades: head injury, anemia, polycythemia vera, polycystic ovarian syndrome, synthetic growth hormones, vitamin A, family history, etc. 

In physiology, the production of CSF occurs in the choroid plexus lining the ventricles. CSF is produced through a Na^+^/K^+^ ATPase channel transporting Na+ to the CSF space with osmosis of H2O through aquaporin-1 channels. Carbonic anhydrase enzyme is vital in the Na^+ ^passage through the production of protons and bicarbonate ions. Then, the CSF flows through the natural ventricular pathway and absorbed by the arachnoid granulations. In the arachnoid granulations, the CSF passes via flow dependent vacuoles determined by the pressure-gradient [14]. 

The primary hypotheses in IIH are centered around an increased resistance to CSF absorption. MRI dynamic phase contrast showed decreased jugular flow in IIH along with altered spinal cord compliance. With the prospects of decreased venous flow, increased research focuses on the pathology of the venous outflow system. Increased ICPs is a known entity in venous sinus thromboses and dural arteriovenous fistulas and can cause some potentially life-threatening symptoms [15]. The first to suggest venous stenosis was King through a diagnostic angiogram, venography, and manometry. In subsequent studies, IIH patients had a significantly higher incidence of bilateral sinus stenosis (or unilateral sinus stenosis with a hypoplastic contralateral sinus) [14]. Though MRV is an acceptable test for venous stenosis, a diagnostic angiogram and venogram is the gold-standard. A 355-patient study on the prevalence of venous stenosis in the general population found a significantly smaller venous diameter (0.7 cm) in females as compared to males [16]. If there is increased resistance to CSF absorption, then the ICP must increase to create a sufficient pressure gradient and overcome the resistance. 

Though there is controversy over whether venous stenosis is a primary cause of IIH or a secondary manifestation of the IIH, Buell et al. added valuable in vivo evidence to push the pendulum towards venous stenosis as a secondary manifestation of IIH. A lumbar puncture was done while measuring in vivo trans-stenosis maximal venous pressures (MVP); CSF drainage revealed a rapid resolution of the pressure gradient and venous stenosis. However, this was short-lived, and the patient eventually needed permanent venous stenting. The hemodynamics between the positive feedback of venous stenosis causing worsening IIH is evident; the increased pressure gradient causes lower venous sinus-CSF gradient, eventually resulting in decreased CSF resorption [17]. This will be re-visited further in the upcoming sections. 

Also, obesity and IIH have been closely linked. It has been proposed that increased abdominal girth increases intra-abdominal pressure, subsequently increasing the venous pressure of the spine and thus the ICP [18]. Though interesting, this hypothesis has not yielded any clinical results. Another theory postulates that IIH is a manifestation of the pro-inflammatory effects of the cytokines secreted by adipose tissue. The increased inflammation is thought to scar the arachnoid villi of the dura, resulting in overall decreased CSF absorption [14].

## Review

Review of non-stenting treatments

Medical Treatments

The first-line treatments for IIH focus on lifestyle and pharmacological interventions. Initially, treatment focused on combating the association of IIH with obesity. As discussed in the pathophysiology section, a clear pathway has not been established but the association is undeniable. The incidence is significantly higher in obese patients and pre-stenotic MVPs in IIH patients, if used as a proxy for ICP, were found to have a direct relationship between BMI and MVP [19]. Thus naturally, weight loss was the first treatment explored for IIH therapy. In a 58-patient study by Kupersmith et al. in 1998, modest weight loss of >2.5 kg improved papilledema by one grading level. Unfortunately, it did not measure headaches or tinnitus and did not have significant improvement in visual acuity or fields [20]. A more invasive study by Sugerman et al. in 1995 explored IIH resolution in morbidly obese post-gastric bypass patients with a mean of 57 kg weight loss. It revealed promising results with a decrease in ICPs from 35 cmH_2_O to 17 cmH_2_O at three years in eight patients [21]. Overall, weight loss has shown to be a promising therapy for IIH. 

The pharmacological approach to IIH has been targeting the choroid plexus to decrease CSF. Acetazolamide has been the mainstay of pharmacological treatment since 1974; McCarthy and Reed showed decreased CSF flow with inhibition of choroid plexus carbonic anhydrase [7]. Recently published, the IIHTT study, a multi-centered randomized double-blinded trial, revealed an improvement in papilledema and visual acuity with high-dose acetazolamide (4g/day) and an improvement in headaches in the subset with mild papilledema. However, this is confounded by the weight loss noted in both blinded groups. An open-arm is currently ongoing and hopefully will shed light in the near future. Also, furosemide has been used in some refractory cases after modest results obtained in a rabbit trial and considered a possible target [22]. In combination, acetazolamide with dietary restrictions constitutes the first-line treatment for IIH.

Surgical Interventions

Alternatively, surgical interventions focus on decreasing ICPs through CSF diversion. In the least invasive approaches, serial lumbar punctures have been used for symptomatic control. However, though vital for diagnosis, the therapeutic utility of repeat symptomatic control has been controversial, increasing the risk of meningitis, arachnoiditis, intracranial hypotension, and tethering patients to repeated painful procedures. The procedure usually gives patients immediate but short-lived relief. CSF should be rapidly replaced in the subarachnoid spaces, but it did not; in the setting of improved venous stenosis with a high volume lumbar puncture, the prolonged relief could be from temporarily normalized CSF outflow, until the stenosis reoccurs [17,23].

Optic nerve sheath fenestration (ONSF) is another form of CSF diversion that aims to prevent the most serious morbidity, visual loss. A window or a series of small slits are made in the retrobulbar optic nerve sheath to drain CSF and decrease the optic nerve edema to prevent visual loss. Even if performed in a single eye, the contralateral eye has been shown to have an improvement in papilledema suggesting that the primary physiology might involve local decompression of the subarachnoid space. Though meant to primarily for the preservation of ophthalmologic function, it has shown to improved headache in ~50% patients [7]. However, the efficacy of the procedure is short lived. At one-year and three-year follow-up, ONSF has significant failure rates of 34% and 45%, respectively [24]. Complications include retinal artery occlusion, neuropathy, hemorrhage, or ophthalmoplegia [9].

The most common form of CSF diversion in IIH is CSF shunting through either a Ventriculoperitoneal shunt (VPS) or a Lumboperitoneal shunt (LPS), with 4,480 performed for IIH between 2005-2009. Historically, LPS has been the preferred choice of CSF shunting due to the technical difficulty of placing a VPS into slit-like ventricles. However, with the advent of image guidance, VPS is becoming the favored method of CSF shunting. VPS has shown to have fewer revisions, decreased shunt migration, shorter hospital stays, and the ability to place programmable shunt valves than an LPS. Also, it avoids the unique possibility of acquired Chiari malformations associated with LPS [25]. Both methods are effective, providing visual stability in 90% of patients [7]. Yet, VPS and LPS still carry significant infectious/revision rates of 30% and 60%, respectively [24].

Venous sinus stenting

King et al. (1995) were the first to describe the venous stenosis through venography and manometry in IIH but Higgins et al. became the first to stent the venous sinus in 2002 on a female with medically refractory IIH [26-27]. Venography revealed bilateral transverse sinus stenosis and after stenting of one side, there was a significant improvement in trans-stenosis gradient and symptomatic control. The pathophysiology characterizing improved CSF resorption through the arachnoid villi after a decrease in venous stenosis has not been proven. Recently, Buell et al. showed improvement of the stenosis and decreased trans-stenosis pressure gradient, though transient, with a high volume lumbar puncture [17]. This would suggest that the venous stenosis is a secondary manifestation of IIH. Yet, the efficacy of the procedure could stem from the prevention of the positive feedback loop and break in the starling-like resistor effect [28]. The inability of the sinus to collapse decreases the distal MVP. This allows for higher trans-arachnoid villi gradient and increases CSF resorption. In theory, the sustained cerebral outflow could buffer against drastic increases in ICPs.

Prior to further discussion of venous stenosis in the setting of IIH, it is important to detail the incidence of venous stenosis in the general population. Durst et al.reviewed a total of 355 normal CT angiographies without structural abnormalities to define normal venous sinus anatomy [16]. Stenosis, as previously defined by Marmarou et al., was an acute reduction in the caliber of the vessel by at least 40%, while a hypoplastic sinus is decreased in average diameter of 40% compared to the dominant venous sinus [29]. Not surprisingly, the male sinuses were larger on average throughout the cerebral venous system. The male to female values in the superior sagittal sinus (SSS), right transverse sinus (RTS), and left transverse sinus (LTS) were 5.21 mm vs 4.57 mm, 6.30 mm vs 5.57 mm, and 5.46 mm vs 4.66 mm, respectively. The arachnoid granulations were present in the SSS in 50% of patients with a 7% incidence of SSS stenosis. Due to the increased prevalence of RTS dominance, it is larger on average than LTS. Most of the stenosis in the sinuses were due to the arachnoid granulations (77% on LTS and 71% on RTS). Overall, only 6% had decreased flow through both sinuses either by bilateral transverse sinus stenosis or unilateral stenosis with contralateral hypoplastic sinus. Contrary to non-IIH patients, bilateral transverse sinus stenosis is prevalent in 90% of IIH patients [30]. Due to the association of stenosis with arachnoid granulations, they could be one of the etiologies of stenosis in IIH patients. Also, the smaller caliber of sinuses could predispose females to increased risk of venous stenosis [16].

Technique

**Figure 2:**
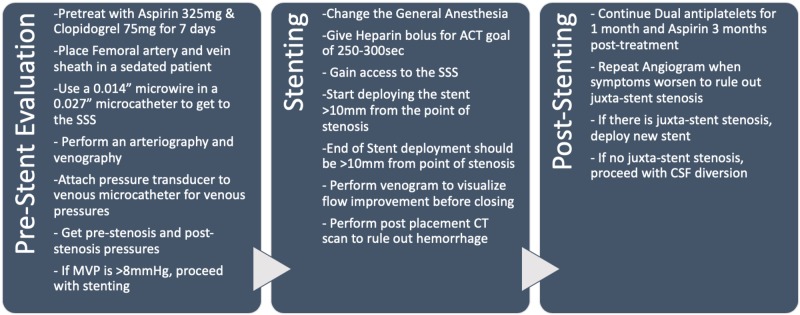
The table summarizes the authors' technique for venous stenting after the initial evaluation and diagnosis of venous stenosis ACT: activated clotting time, SSS: superior sagittal sinus

In venous sinus stenting (VSS), preoperative evaluation is paramount in the selection of the right patient population. All IIH patients should be assessed by neuro-ophthalmology for papilledema, visual acuity, optical coherence tomography, and visual field testing. MRI should be done with possible MR angiography and MR venography in appropriate circumstances. Elevated ICPs should be defined through either a high volume lumbar puncture or intracranial pressure monitor. The authors’ preferred method of treatment is to perform a cerebral angiography, venography, and venous manometry under conscious sedation. Cerebral angiography is performed with a guide catheter through a femoral arterial puncture. Cerebral venography and manometry are done through femoral vein access, with a shuttle catheter placed at the internal jugular vein. Then, a 0.027-in microcatheter with a 0.014-in microwire is used to gain SSS access past the point of stenosis. Then, an arterial pressure transducer is zeroed and pressures are measured from the segments of SSS, bilateral transverse sinuses, sigmoid sinus, jugular bulb, and cervical internal jugular. Patients with elevated MVPs or significant trans-stenosis pressure gradient (>8 cmH_2_O) are offered a venous stent. Ultimately, venous manometry is a decision-making factor for VSS. Prior to stent placement, patients are given seven days of Aspirin 325 mg and Clopidogrel 75mg daily. Therapeutic P2Y12 (<208) and ARU (620-672) levels are needed to proceed or the patients are loaded with aspirin 325mg and clopidogrel 600mg. The stent placement procedure is done under general anesthesia for patient comfort. Venous access is attained and heparin bolus is given for a goal-activated clotting time of 250-300 seconds through the procedure. The access sheath system usually used is a triaxial construct of 12F, 9F, and 7F sheaths of length 80-90 cm. MVPs and trans-stenosis pressure gradient are reconfirmed prior to placement of the venous stent. The choice of a stent may vary based on individual patient anatomy. The stent is placed to span 10-mm pre-stenosis and post-stenosis. Post-stenting venograms are performed to look at the drainage patterns. The procedure is completed by closing the venous access by pressure. A CT scan is performed to rule out an intracranial hemorrhage. Patients will be on dual antiplatelet therapy for one month and aspirin 325 mg for three months. They will have a repeat angiogram at three months to evaluate for any complications [31]. The complete technique is summarized in Figure 2. The procedure is evolving with increasing experience and has been improved from the initial iterations [32].

Intracranial Pressures and Pressure Gradient

The goal of the venous stenting has been to normalize the trans-stenosis pressure gradient. A pressure gradient of 8-10 cmH_2_O has been the threshold for intervention in most studies [31-34]. The first large cohort study (*n *= 12) by Higgins et al. in 2003 showed a decrease in the mean pressure gradient from 18.9 to 11.3 cmH_2_O [35]. A larger 185-patient review revealed a decreased pressure gradient from 20.1 cmH_2_O to 4.4 cmH_2_O. The initial CSF opening pressure was 35.7 cmH_2_O (95% CI, 34.8 cmH_2_O to 36.2 cmH_2_O) but unfortunately was not able to determine the post-stent CSF pressure [24]. In the first prospective study of VSS for IIH, the author and his colleagues proved correlation of intracranial ICP (mean: 42.2 +/- 13.2, range 20-73) to maximum venous pressure (39.5 +/- 14.9, range 22-68) in a cohort of 10 patients. Also, the pre-stent pressure gradient mean of 30.0 cmH_2_O resolved to post-stent pressure gradient mean of 1 cmH_2_O. Resolution of ICPs was also proven with post-stent ICP mean of 17.0 cmH_2_O [31]. Within the studies, many patients had significant symptomatic relief even with <8 cmH_2_O gradient. 

Resolution of Symptoms

VSS is still a novel treatment and not used as a first-line treatment; thus, the current data are in medically refractory IIH patients. Higgins and colleagues’ initial report improved headaches, vision, and papilledema in seven of 12 patients. But Higgins’ study suffered from poor patient selection as only three patients underwent pre-stent LP and ~66% had papilledema [35]. As more studies became available, venous stenting showed great results for symptomatic control of IIH. In the 185-patient review, 78.3% improved headaches and 92.9% improved tinnitus over a 22.2-month average follow-up [32]. The largest retrospective cohort study available by Ahmed et al. showed a 93% (40/43 patients) improvement in headaches and 100% improvement in tinnitus [33]. The author’s cohort of 50 patients revealed a 67.7% improvement in headaches and 46.7% improvement in tinnitus. In addition, only 3%, 10%, and 3.3% had worsened in the same categories, respectively. This data is a pending publication [19]. The prospective cohort study revealed a 90% improvement in headaches [31].

Ophthalmologic Outcomes

Current data are especially promising for the ophthalmologic outcomes. Higgins’ study had the worst outcomes for papilledema with only a 62.5% improvement [35]. However, excluding that data set from the 185-patient review, 79/81 patients (97.5%) showed improvement in their papilledema. Similarly, 57/62 patients (92%) improved their vision at follow-up [24]. A prospective study showed promising results. Seven of the seven patients with papilledema pre-stenting had a resolution at six-month follow-up on funduscopic imaging and optical coherence tomography. 50% of patients had improvement of vision, while another 50% had a stable vision [31]. In addition to visual fields, a recent report by Ding et al. demonstrated the rapid correction of bilateral abducens nerve palsies within eight hours of stenting [36].

Complications

With any vascular stenting, the most feared complication is acute thrombus and stenosis of the stent. This would increase the distal venous pressure and cause either an ischemic or hemorrhagic stroke. Fortunately, there has not been a recorded thrombosis in VSS, even with the use of bare metal stents. Another complication of stents is acute re-stenosis of the stent. Atrial stents are known to collapse within the stent, referred to as in-stent stenosis. However, stenosis in VSS affects the sinus adjacent to the stent, referred to as stent-adjacent stenosis (SAS); this has been documented at the distal end of the stent. The incidence is ~18% of venous stents, but are symptomatic and/or require retreatment/extension in ~10% of times [37]. The review by Starke et al. found a 3.5% incidence of in-stent stenosis but one alone required retreatment. The in-stent stenosis occurred in the Higgins cohort and required thrombolytic therapy. However, the protocol only used anticoagulation as opposed to the now standard dual-antiplatelet therapy. SAS is more frequent with an incidence and retreatment rate of 11.4% and 6%, respectively. Currently, there are no set criteria for retreatment. Interventionists have either elected to treat the SAS upfront to prevent the reoccurrence of the positive feedback loop or selective treatment for symptomatic patients [24]. The repeat procedure would add additional stents to expand the level of new stenosis. In the prospective cohort, two of the 10 patients experienced SAS. One was treated immediately due to the reoccurrence of symptoms and the presence of a treatable pressure gradient. The other did not initially complain of symptoms, but follow-up angiogram revealed a significant pressure gradient of 48 cmH_2_O and 65% stenosis; thus, it was treated [31].

As the most common complication of VSS, initial classifications have sought to predict the occurrence of SAS. Raper et al. proposed a three-type classification system [37]. It categorized the pattern of trans-stenosis pressure gradient resolution according to the change of the venous pressures following the stenting. Type I pressure gradient change has a reduction of the distal MVPs with stable proximal MVPs. Type II pressure gradient change has an increase of the proximal MVPs with unchanged distal MVPs. Lastly, Type III pressure gradient change has a reduction of the distal MVPs and increase in the proximal MVPs. Of 47 patients retrospectively reviewed, type I had significantly lower rate of SAS requiring re-treatment (0%) than type II (28.6%) and type III (22.7%; *p *= 0.018 & *p *= 0.031, respectively). 

Other complications include femoral pseudoaneurysm (*n *= 2), subdural hematoma (*n *= 3), transient hearing loss (*n *= 2), and stent migration (*n *= 1). Overall, the rate of other adverse effects is 5.4%. A few case reports have discussed individual events [24]. Lavoie et al. reported severe cerebellar hemorrhage after transverse sinus stenting not associated with sinus occlusion. They hypothesized that the microware or stent caused a dural tear in the sinus wall and caused the hemorrhage [38]. Buell et al. reported a unique case of intracranial dural arteriovenous fistula (dAVF) formation after long construct VSS from the SSS to the right sigmoid sinus. On repeat angiogram, a *de novo* Borden type I dAVF was found draining into the right transverse sinus (TS)/sigmoid sinus (SS) junction. The stenting may have caused hemodynamic changes that resulted in decreased cortical outflow and formation of the dAVF; the VEGF and PDGF expression secondary to VSS-induced inflammation may have promoted the vascular proliferation. It was a low-grade dAVF and thus treated conservatively [39].

Studies have examined the changes to venous patterns secondary to venous stenting, mainly at the vein of Labbé (VOL). Naturally, the VOL drains into the distal transverse sinus or TS/SS junction. Venous stents often span the ostium of VOL. A recent series by Levitt et al. report that the venous stenting does not change the overall venous drainage of the VOL [40]. Another retrospective study by the University of Virginia group found altered flow ~25% immediately after stenting and ~26% on follow-up angiography [41].

Body Mass Index

The relationship between obesity and IIH has been well established; however, the efficacy of VSS stratified by BMI has just started being explored. Raper et al. retrospectively categorized 50 patients by BMI categories to examine if VSS is equally effective in different BMI groups. In conclusion, it identified a small but direct relationship with BMI and MVP. As for the efficacy, none of the symptoms revealed a statistically significant difference and noted VSS was equally effective throughout the BMI categories. The percentage reduction in the pressure gradient post-stent is similar between the BMI categories (~83%) [19]. 

Conscious versus General Anesthesia

As VSS becomes more prevalent, various practice differences are emerging between interventionists. From the studies on ICPs, there is a difference between cranial pressure measurements while under conscious sedation (CS) and general anesthesia (GA). These authors, unless in extenuating circumstances, base the treatment decision on the trans-stenosis pressure gradient under CS. A 61-patient retrospective cohort that underwent venous pressure measurement under CS and GA revealed a small but significantly lower MVP under GA (19.8 cmH_2_O) than CS (21.9 cmH_2_O; *p* = 0.029). The MVPs lowered in the distal venous sinuses and increased in the proximal venous sinuses under GA. This significantly lowered the pressure gradient from a mean of 12.1 cmH_2_O under CS to 8.9 cmH_2_O under GA (*p *< 0.001). A subset, however, of patients had their pressure gradient increase under GA and were more likely to normalize after VSS [34].

Future directions

*Stent Type* 

Currently, there is no consensus on the stent of choice. For VSS, the stents need to be self-expanding with adequate radial force to overcome any external stenosis from elevated ICPs and long constructs to ensure they extend >10 mm pre- and post-stenosis. Review of the literature includes an array of stents initially meant for biliary and peripheral artery lumens, Zilver 518 of 10-mm diameter (Cook Medical), SMART 10-mm diameter stent (Cordis), Protégé Everflex (Covidien), Precise (Cordis), and Wallstent (Boston Scientific) [31-32]. Future studies should seek to find and optimize constructs that would prevent sinus rupture, prevent thrombosis, and reduce SAS. 

Anticoagulation

Since the start of VSS, there have been constant changes in the anticoagulation protocol used throughout the various studies. All the recent studies ensure the patients are dual anticoagulated appropriately prior to the VSS. This usually requires testing of the P2Y12 and ARU levels. Yet, that is where the similarities end; most stop clopidogrel within the first six months while aspirin recommendations range from three months to a lifetime [42]. The practice is derived from the arterial stenting practices since borrowed iliac and biliary stents are used for the stenting. Also, the risk of spontaneous hemorrhages is presumed to be lower in the younger patients. However, antiplatelet and anticoagulation are not routinely recommended in other venous stents, since as an inferior vena cava filter [43]. In addition, a traditional IIH patient is a female in her 20s and 30s with potential for pregnancy. Overall, anticoagulation in the setting of VSS is an area that warrants further evaluation.

Expanding Venous Stenting

The success of the VSS has been largely due to the careful patient selection. It has proven to be great therapy for IIH patients. As it becomes more commonplace, there will be a search for new and expanding indications. A study by Raper et al. evaluated the safety and efficacy of SSS stenting in non-thrombotic venous occlusive disease. Of the 19 patients reviewed, 58% were *de novo* stents while 42% had prior transverse sinus stents. By dividing the SSS into four equal segments, Raper and colleagues found the stenosis at S1 segment, closest to the torcula, was more frequent (~93%). Patient symptoms did resolve after SSS stenting; headache, tinnitus, and visual obscurations improved by 66.7%, 63.6%, and 50%, respectively. However, the mean trans-stenosis pressure gradient was only 4.2 cmH_2_O in this cohort and then decreased to 1.5 cmH_2_O post-stenting [44]. Drastically less than the 8 cmH_2_O used in the TS-SS segments, this illustrates the importance of patient selection. There are patients who would benefit from VSS outside of IIH, but the difficulty could be in identifying them. In a 250-patient pilot questionnaire by Chandran et al., 81% enrolled in a randomized trial between VSS and CSF shunt. 

Ehlers-Danlos Syndrome, Hypermobility Type

Ehlers-Danlos syndrome, hypermobility type (hEDS; EDS type III) is a hereditary connective tissue syndrome with an unknown genetic mutation diagnosed exclusively by clinical assessment and family history [45]. Patients with EDS type III have joint hypermobility, tissue fragility, and skin extensibility and present with headaches and chronic fatigue [46-47]. These headaches have been closely linked to venous hypertension. In a dataset pending publication, a 130-patient cohort showed a female predilection of 10:1 while presenting with headaches, tinnitus, and visual abnormalities. Intracranial hypertension was discovered in 30% of the cohort through radiographic imaging. Data analysis revealed that symptomatic relief can be achieved with a smaller trans-stenosis pressure gradient (*p *< 0.0001) in about 77% of the cohort. However, this cohort had a higher incidence of junctional stenosis (~11%). This initial report suggests EDS type III could be another indication for intracranial VSS.

## Conclusions

Conclusion

Venous sinus stenting is an increasingly utilized technique for venous pathology (i.e .IIH). IIH is a disorder defined by classic symptoms (headaches, vision loss, and tinnitus) and elevated intracranial pressure without an identifiable source. The current collection of studies provides mounting evidence that VSS provides symptomatic relief as well as possible disease control. Additional follow-up and further studies are necessary to provide prospective and prolonged data to evaluate the durability of VSS as well as long-term clinical outcomes. Overall, VSS has proven to be valuable adjuvant for elevated intracranial pressure and could be used for more venous pathology.
